# Antibiofilm and antipersister activity of acetic acid against extensively drug resistant *Pseudomonas aeruginosa* PAW1

**DOI:** 10.1371/journal.pone.0246020

**Published:** 2021-02-02

**Authors:** Madhumita S. Tawre, Ekta E. Kamble, Shital N. Kumkar, Mansura S. Mulani, Karishma R. Pardesi

**Affiliations:** Department of Microbiology, Savitribai Phule Pune University, Pune, Maharashtra, India; Laurentian University, CANADA

## Abstract

*Pseudomonas aeruginosa* is an ESKAPE pathogen associated with difficult-to-treat burn wound and surgical-site infections. This study aimed to characterise an extensively drug resistant (XDR) *P*. *aeruginosa* isolate (designated PAW1) and to investigate the antibiofilm and antipersister effect of acetic acid on PAW1. PAW1 was identified using biotypic (VITEK) and genotypic (16S rDNA) analysis. Minimum inhibitory concentration (MIC) and disc susceptibility testing showed high level resistance against all antibiotics from classes including beta lactams, cephems, carbapenems and fluoroquinolones. It was therefore identified as extensively drug resistant (XDR), showing resistance to all antibiotics except for, aminoglycoside (gentamicin and netilmicin) and lipopeptides (polymyxin B). Time kill assays showed antibiotic tolerant, persister cell formation in presence of 100X MICs of gentamicin and polymyxin B. Other virulence traits such as ability to produce lipase, protease, haemolysin, and siderophores and to form biofilms were additional factors which may contribute to its pathogenicity. PAW1 showed promising susceptibility against acetic acid with MIC and minimum biofilm inhibitory concentration of 0.156% (v/v). Percent viability of PAW1 was dependent on dose and treatment time of acetic acid. 0.625% acetic acid treatment of 5 minutes was effective in killing >90% planktonic cells showing lesser toxicity to L929 cells (IC_50_ = 0.625%). Biofilm disruption caused due to acetic acid was also dose dependent, showing 40.57% disruption after treatment with 0.625% acetic acid for 5 minutes. FESEM imaging and live dead staining of planktonic and biofilm forms of PAW1 confirmed that acetic acid treatment caused 19.04% of cell shrinkage and disruption of extracellular matrix resulting in killing of cells. Antipersister activity of acetic acid was demonstrated by showing complete killing of PAW1 at 4X MIC. Overall, this study characterised an XDR isolate *P*. *aeruginosa* showing resistance and tolerance to various antibiotics. Antipersister and antibiofilm effect of acetic acid demonstrates the importance of forgotten topical agents as an effective strategy to treat XDR pathogens.

## Introduction

The acronym “ESKAPE” denotes six hospital associated pathogens: *Enterococcus faecium*, *Staphylococcus aureus*, *Klebsiella pneumoniae*, *Acinetobacter baumannii*, *Pseudomonas aeruginosa*, *and Enterobacter spp*. with formidable and ever-present adversary associated with broad spectrum of infections in humans [1, 2]. *P*. *aeruginosa* is one of the ESKAPE members particularly associated with secondary wound infections which are difficult to treat owing to the multidrug resistant (MDR) nature of this pathogen further narrowing down the usage of existing antibiotics, topical antiseptics and disinfectants [3].

Besides multidrug resistance, *P*. *aeruginosa* also has virulence traits such as biofilm forming ability, siderophore production, production of extracellular enzymes and other proteins responsible for pathogenicity [4, 5] which add to the arsenal available to cause infections. Moreover, *P*. *aeruginosa* is also known to enter a persister state during which a sub-population of cells remains metabolically inactive, therefore tolerating the presence of antibiotics [6]. This persister state is defined by the ability of an organism to survive high doses of an antibiotic despite being sensitive to it at lower concentrations. *In vitro* treatment of a population of antibiotic sensitive cells to high doses of the antibiotic, typically results in a biphasic curve where there is an initial decline in the number of cells followed by a plateau that represents persister cells [7]. Both, biofilm forming ability and persister state are a major cause of the recurrent infections caused by this pathogen [8–11].

Such a disquieting scenario drives towards the development of new drugs along with investigation of alternative strategies for eradication of such pathogens. Alternative therapies that appear effective against MDR pathogens include, bacteriophage therapy, photodynamic light therapy, use of antimicrobial peptides, use of metal nanoparticles, and combination drug therapy (antibiotic-antibiotic and antibiotic with other antimicrobial agents). However, most of these therapies are still in stage of infancy, thus may take time to be translated into clinical practice [2, 12].

Topical antiseptics and disinfectants such as chlorhexidine, dettol, povidone-iodine are commonly used in hospitals to treat pseudomonal wound infections [13]. Organic acids such as acetic acid, ascorbic acid, salicyclic acid, citric acid, boric acid and lactic acid also offer an effective means to eradicate *P*. *aeruginosa* from skin infections when applied topically [13, 14]. Renaissance of acetic acid dressings serves as the Holy Grail for the infected wound management [15, 16]. With the forbidding circumstances of antibiotic resistance, forgotten antimicrobials such as acetic acid should come in to play.

There are very few reports studying effect of acetic acid on biofilms formed by *P*. *aeruginosa* [17, 18]. To the best of our knowledge there are no studies showing if persister cell formation occurs in presence of acetic acid. The present study therefore aimed to characterise an extensively drug resistant *P*. *aeruginosa* strain isolated from a chronic sternal wound infection and to determine susceptibility of its planktonic and biofilm forms towards acetic acid.

## Materials and methods

### 1. Isolation and identification of the pathogen

*P*. *aeruginosa* (designated as PAW1) used in the present study was an isolate from a recalcitrant sternal wound infection of a diabetic patient who was put on negative pressure wound therapy. The identity of the isolate was confirmed by biochemical identification using GN cards on VITEK 2 system (Biomérieux, France). Genotypic identification was done by 16S rDNA sequencing using universal primers 8F and 1492R. Amplicons were sequenced using the ABI BDT v3.1 cycle sequencing kit on the Illumina platform. BLAST was carried out on the BlastN site at the NCBI server. A phylogenetic tree depicting the relatedness of PAW1 with type strains of *P*. *aeruginosa* ATCC 10145, *P*. *syringae* ATCC 19310 and *Staphylococcus aureus* ATCC 12600 was constructed by the neighbor joining tree method using Mega7 based on the bootstrap test of 1000 replicates. The isolate was maintained in Luria Bertani (LB) broth unless otherwise stated and stored in glycerol stocks for further characterization.

### 2. Determination of virulence traits of PAW1

#### 2.1. Resistance to antibiotics

Resistance of PAW1 to the 20 antibiotics recommended by Clinical and Laboratory Standards Institute (CLSI, USA, 2018) was tested using antibiotic disc susceptibility method. Antibiotics used and their disc concentration are as listed in Table 1. Minimum inhibitory concentration (MIC) of antibiotics against PAW1 was determined by broth micro dilution method using concentrations ranging from 2–1024 μg/mL (CLSI, USA, 2018). *P*. *aeruginosa* NCIM 5029 (ATCC 27853), *Escherichia coli* NCIM 2931 (ATCC25922) and *S*. *aureus* NCIM 5021 (ATCC 25923) were used as reference strains for the study.

**Table 1:** Zone diameter and MIC values of CLSI recommended 20 antibiotics against PAW1.

Test Group	Disc concentration	Zone diameter	MIC
Antimicrobial agent	(μg/disc)	(mm)	(μg/mL)
**Penicillins**			
Piperacillin	100	0^R^	128^R^
**B-Lactam Inhibitor Combinations**			
Piperacillin-tazobactam	100/10	10^R^	128^R^
Ticarcillin-clavulanate	75/10	0^R^	>1024^R^
**Cephems**			
Ceftazidime	30	0^R^	>1024^R^
Cefepime	30	0^R^	>1024^R^
**Monobactams**			
Aztreonam	30	20^I^	16^I^
**Carbapenems**			
Doripenem	10	0^R^	1024^R^
Imipenem	10	0^R^	1024^R^
Meropenem	10	0^R^	1024^R^
**Lipopeptides**			
Colistin	10	16^R^	16^R^
Polymyxin B	300U	**16**^**S**^	**≤2**^**S**^
**Aminoglycosides**			
Gentamicin	10	**21**^**S**^	**≤2**^**S**^
Tobramycin	10	12^R^	64^R^
Amikacin	30	14^R^	64^R^
Netilmicin	30	**23**^**S**^	**≤2**^**S**^
**Fluoroquinolones**			
Ciprofloxacin	5	0^R^	256^R^
Levofloxacin	5	0^R^	512^R^
Norfloxacin	10	0^R^	512^R^
Ofloxacin	5	0^R^	>1024^R^
Gatifloxacin	5	0^R^	256^R^

Note: MIC breakpoints as per CLSI 2018 guidelines were used to define susceptible (S, indicated in bold text), intermediate (I) and resistant (R) to 20 antibiotics; 0 signifies no zone of inhibition.

#### 2.2. Tolerance to antibiotics

PAW1 was found to be susceptible to polymyxin B (MIC = 0.025 μg/mL) and gentamicin (MIC = 1 μg/mL). Generation of antibiotic tolerant persister cells in a stationary phase planktonic cell population of PAW1 was determined using the time kill assay. Briefly, a single colony of PAW1 was inoculated in 2.25 mL LB broth. The tubes were incubated for 16–18 h to obtain a stationary phase culture. To this, antibiotics were added at 100X MIC and 100 μL samples were retrieved at hourly intervals for 9 h and one after 24 h. The samples were washed with saline, diluted and then spot inoculated on LB agar plates to quantify the surviving population. The experiment was conducted in triplicates. Time kill curve against each of the antimicrobial was plotted to check for the characteristic biphasic curve. The experiment was carried out thrice in duplicates.

#### 2.3. Production of extracellular enzymes, haemolysins and siderophores

The proteolytic activity of PAW1 was assessed on gelatin agar and skimmed milk agar medium. Caseinase activity using 1% azocasein (Sigma-Aldrich, Germany) as substrate was determined as reported earlier [19]. Lipolytic activity of cell free supernatant of PAW1 was determined using p-nitrophenyl palmitate as the substrate [20]. Haemolysin and siderophore production ability of PAW1 was examined qualitatively by spot inoculating an overnight grown culture on 7% sheep blood agar (HiMedia, India) [21] and chrome azurol S (CAS) agar [22] respectively.

#### 2.4. Biofilm formation assay

Quantification of biofilm synthesized by PAW1 was carried out as described earlier [11, 23] with some modifications. Briefly, O.D. adjusted culture was inoculated in LB broth in a microtitre plate to achieve C.F.U. of 10^5^ cells/mL and incubated at 37°C for 24 h. Culture was removed and the wells were washed thrice with phosphate buffered saline (PBS). Crystal violet (0.4% w/v) was then added to the air-dried wells and incubated for 15 min at room temperature. After washing the wells under a gentle stream of water and air drying them, the dye bound to the biofilm was solubilized in 33% (v/v) acetic acid and absorbance was measured at 630 nm using microplate reader (Spectra Max M2, Molecular Devices, USA). *P*. *aeruginosa* NCIM 5029 (ATCC 27853) and *E*. *coli* NCIM 2931 (ATCC 25922) were used as positive and negative controls respectively. The experiment was carried out in three biological replicates. Relative biofilm formation was determined by comparing with the reference strain *P*. *aeruginosa* NCIM 5029.

### 3. Antimicrobial, antipersister and antibiofilm activity of acetic acid on PAW1

#### 3.1. Effect of acetic acid on viability of PAW1

MIC of acetic acid against PAW1 was determined by broth micro dilution method. Briefly, cells were seeded in microtitre plates at a density of 10^5^ cells/mL and treated with acetic acid at concentrations ranging from 0.01–5% (v/v) [17]. Plates were then incubated at 37°C for 24 h after which absorbance was recorded at 540 nm. The experiment was carried out in triplicates and the most frequent MIC value was reported.

To further confirm the antibacterial activity of acetic acid on PAW1 cells, MTT dye reduction assay using 3-(4,5-dimethylthiazol-2-yl)-2,5-diphenyltetrazolium bromide (Himedia, India) was performed [24]. Briefly, 150 μL culture (10^5^cells/mL) was treated with acetic acid at concentrations ranging from 0.039–5% for 2.5, 5.0, 7.5 and 10 min. Treated cells were immediately washed with PBS, and incubated with 150 μL of MTT solution (0.5 mg/mL) in dark for 4 h at 37°C. The resulting formazan crystals were dissolved in 150 μL DMSO, incubated for 20 minutes and absorbance read at 595 nm. Percent viability was determined using the formula

$$Percentage\;viability=\frac{A\;at\;595\;nm\;with\;acetic\;acid\;}{A\;at\;595\;nm\;without\;acetic\;acid}\;X\;100$$

Where A stands for absorbance.

#### 3.2. Tolerance of PAW1 to acetic acid

PAW1 was found to be susceptible to acetic acid with MIC = 0.156%. Generation of persister (tolerant) population in stationary phase planktonic cells of PAW1 was carried out by exposing them to 2X (0.312%) and 4X (0.625%) MIC concentration of acetic acid using the time kill assay as described above. 100 μL samples were retrieved at definite time intervals up to 3 h. They were immediately washed with saline, diluted and then inoculated on LB agar plates to quantify the surviving population. Time kill curve was plotted to check for the characteristic biphasic curve. The experiment was carried out thrice in duplicates.

#### 3.3. Effect of acetic acid on biofilms formed by PAW1

Effect of acetic acid on biofilm synthesis as well as on preformed biofilms was tested using biofilm inhibition and disruption assay respectively. For biofilm inhibition assay, O.D. adjusted culture was inoculated in LB broth containing acetic acid (0.01–5%) in a microtitre plate to achieve C.F.U. of 10^5^cells/mL. Plates were incubated at 37°C for 24 h after which, culture was removed and the wells were washed twice with PBS. Crystal violet assay was performed using the protocol as described above to estimate the amount of biofilm formed in comparison with the untreated control.

For biofilm disruption assay, O.D. adjusted culture was inoculated in LB broth in a microtitre plate to achieve C.F.U. of 10^5^cells/mL. Plates were incubated at 37°C for 24 h to allow biofilm formation. Preformed biofilms were then treated with 0.01–5% of acetic acid for 5 min after which, culture was removed and the wells were washed twice with PBS. Crystal violet assay was performed to estimate the amount of disrupted biofilm by comparing it with the untreated control. Percent biofilm inhibition and disruption were determined using the formula:

$$Percentage\;inhibition\;or\;disruption=\frac{\left(A\;at\;630\;nm\;without\;acetic\;acid-A\;at\;630\;nm\;with\;acetic\;acid\right)}{A\;at\;630\;nm\;without\;acetic\;acid}\;X\;100$$

Where A stands for absorbance.

#### 3.4. Field Emission Scanning Electron Microscopy (FESEM) of acetic acid treated planktonic and biofilm forms of PAW1

Log phase planktonic cells of PAW1 treated with 0.625% acetic acid for 5 min along with untreated control were observed by FESEM (Quanta FEG 450, Netherlands). Briefly, treated cells were gently washed and re-suspended in PBS and 2 μL of this suspension was dropped onto silicon wafer (5 mm x 5 mm). Further, the cells were fixed with 2.5% glutaraldehyde at 4°C and kept overnight followed by PBS wash. Dehydration was done sequentially with grades of ethanol 20, 40, 60, 80 and 90% for 15 min each and then twice with absolute ethanol. Sample was dried and coated with platinum and observed under FESEM [25].

Disruption of pre-formed biofilm was also observed by FESEM. Briefly, biofilm was allowed to form on sterile glass slides (5 mm x 5 mm cut pieces) at 37°C for 24 h. Pre formed biofilm was treated with 0.625% of acetic acid for 5 min. The slides were washed with PBS and processed as mentioned above for FESEM.

#### 3.5. Live/Dead staining of acetic acid treated planktonic and biofilm forms of PAW1

Cell viability was tested using LIVE/DEAD BacLight™ Bacterial viability kit (Invitrogen, California). Log phase planktonic cells of PAW1 treated with 0.625% acetic acid for 5 min along with untreated control were stained as per manufacturer’s protocol. Similarly, 24 h old pre-formed biofilm of PAW1 allowed to form on coverslips, was treated with 0.625% acetic acid for 5 min. After treatment, the cover slips were washed twice with PBS and stained using BacLight™ dye mixture. The cells/ biofilms were incubated for 15 min in dark and then washed with PBS. The fluorescence from both live (green) and dead (red) bacteria was viewed separately using filters with excitation wavelength of 450–490 nm and 545–570 nm (Zeiss Axioscope A1, Germany).

### 4. Cytoxicity of acetic acid against L929 mouse fibroblast cell line

Toxicity of acetic acid was tested on L929 mouse fibroblast cell lines (procured from National Centre for Cell Sciences, Pune, India) using MTT assay. Briefly, L929 cell lines were cultured in Dulbecco’s Modified Eagle’s Medium (Himedia, India) supplemented with 10% fetal bovine serum, streptomycin (0.1 mg/mL) and penicillin (100 U/mL) and maintained at 37°C, 5% CO_2_. Cells were seeded in microtitre plate at a density of 10^4^ cells/mL and incubated for 24 h. The cells were then treated with acetic acid concentrations ranging from 0.039–5% for 2.5, 5, 7.5 and 10 min, immediately followed by aspiration and replacement with fresh medium [26]. After 24 h of incubation, the medium was aspirated and MTT assay was performed as described above. The assay was also carried out using untreated cells as the control.

### 5. Statistical analysis

Results of all experiments were determined as means± SD. One-way ANOVA followed by Tukey’s HSD post hoc test was carried out for time kill assays, biofilm inhibition, biofilm disruption and MTT assays. Differences were considered statistically significant at p< 0.01.

## Results

### 1. Biotype and phylogenetic analysis of the isolate

Preliminary identification revealed that the isolate was Gram negative, oxidase and catalase positive motile rod, identified as *Pseudomonas aeruginosa* (Bionumber 0003453003500000) using VITEK biotyping and confirmed by 16S rDNA analysis (100% identity with *Pseudomonas aeruginosa* ISB4- Accession No. KJ507205, query length 1344 bases, 100% query cover). The consensus sequence of the same is available in NCBI database under accession number MG786591.

### 2. PAW1 demonstrated various virulence traits

PAW1 exhibited proteolytic, haemolytic and lipolytic activities suggesting its ability to induce tissue damage. Caseinase activity of the cell free supernatant was found to be 1.15±0.189 units while lipase activity was 6.92±0.18 μM/min. PAW1 also showed the ability to produce siderophore, an essential iron chelating agent that may be required for its survival and evasion of host immune response. Relative biofilm formation was 100±17% when compared with that of *P*. *aeruginosa* NCIM 5029 (ATCC 27853). Biofilm formation by *E*.*coli* NCIM 2931 was 1.77±0.26%.

### 3. PAW1 was an extensively drug resistant isolate with ability to form persister cells

PAW1 exhibited high degree of resistance to all antibiotics recommended by CLSI except gentamicin, netilmicin and polymyxin B (Table 1). Intermediate to high level resistance was observed for all antibiotics belonging to class penicillins, β-lactam inhibitor combinations, cephems, monobactams, carbapenems, fluoroquinolones with MICs ranging between 128 to ≥1024 μg/mL. It can therefore be concluded that PAW1 is an extensive drug resistant (XDR) isolate as per the criteria defined earlier [27].

**Fig 1:**
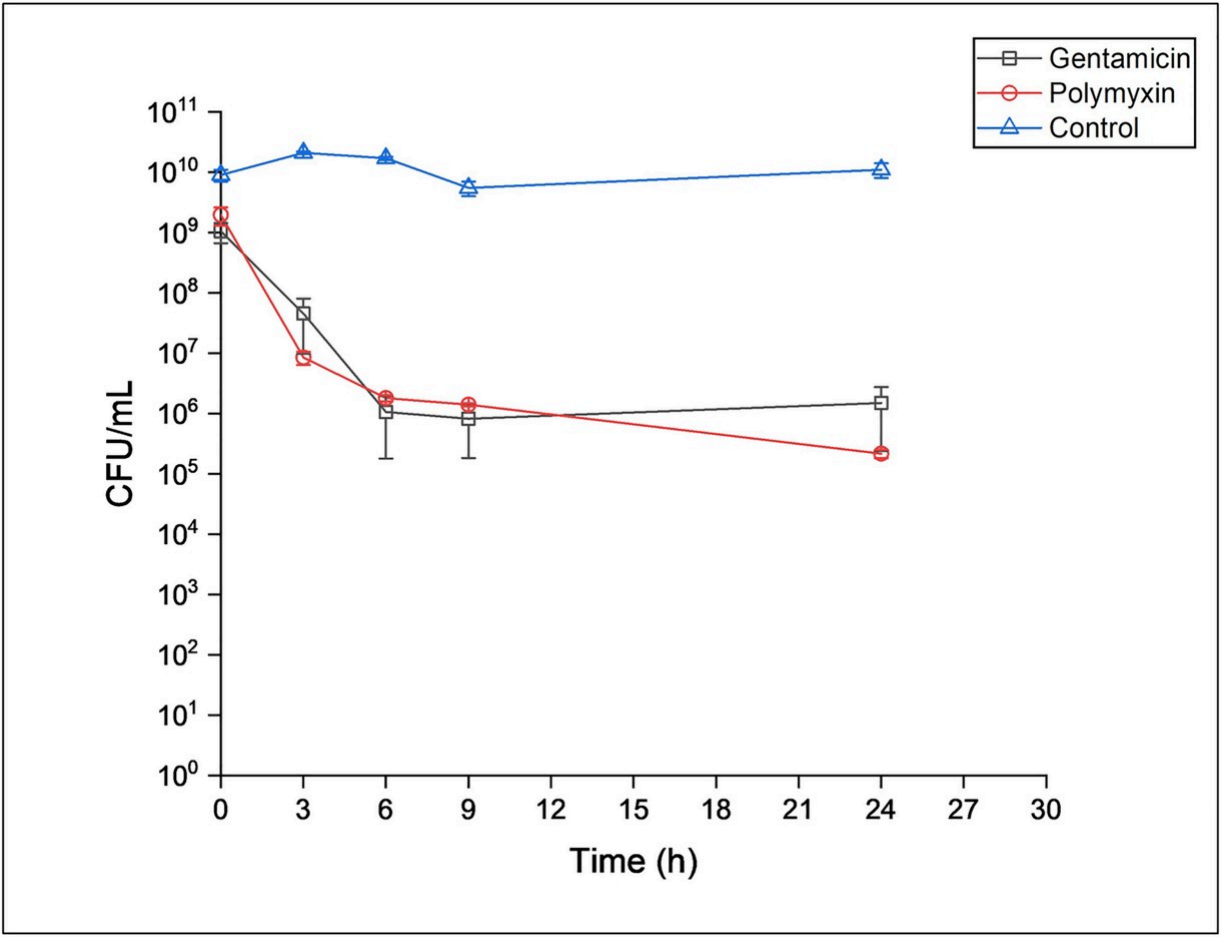
Time dependent killing of stationary phase culture of PAW1 against 100X MIC of polymyxin B and gentamicin.

The time kill curve showed a biphasic pattern typical of persister cells (Fig 1). The stationary phase population of PAW1 was reduced 3 log_10_ by polymyxin B within 3 h and gentamicin within 6 h after which the colony count remained constant till 24 h. The plateau obtained due to the constant C.F.U. represents the surviving population i.e. persister cells in presence of these antibiotics.

### 4. Acetic acid exerts antimicrobial, antipersister and antibiofilm effect on PAW1 with moderate toxicity against L929 host cells

**Fig 2:**
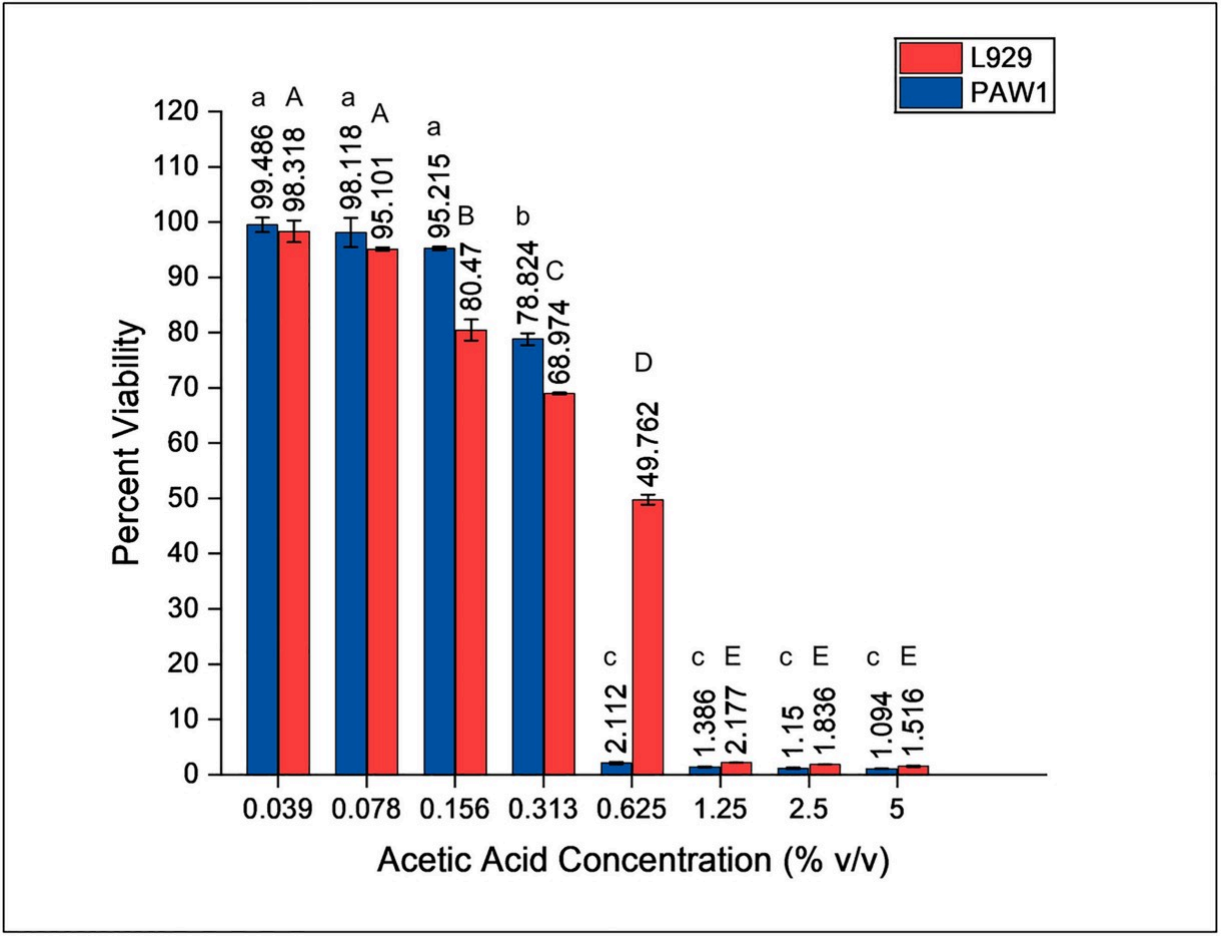
Viability (%) of L929 and PAW1 cells after acetic acid treatment at 0.039–5% (v/v) for 5 min. Error bars indicate standard deviation from three biological replicates. Different letters on the bars indicate that mean values of treatments are significantly different at p<0.01 according to Tukey’s post hoc test.

MIC of acetic acid against PAW1 was found to be 0.156%. Increased exposure time (2.5 to 10 min) and increased doses of acetic acid (0.039–0.625%) were inversely proportional to the percent viability of PAW1 and L929 host cells. Percent viability of PAW1 and L929 at 0.625% acetic acid concentrations was statistically significant (p<0.01) between the various treatment times (S1 and S2 Tables). Only 2.11% (IC_90_ = 0.625%) of PAW1 cells survived at 0.625% acetic acid treatment given for 5 min (Fig 2). Cytotoxicity studies using L929 cells treated at different concentrations of acetic acid for 5 min revealed IC_50_ of 0.625% at which 49.76% cells survived (Fig 2). These results demonstrate the antimicrobial potential of acetic acid which at a dosage of 0.625% (4X MIC) treated for 5 minutes resulted in effective killing of PAW1 cells causing 50% toxicity to L929 cells.

**Fig 3:**
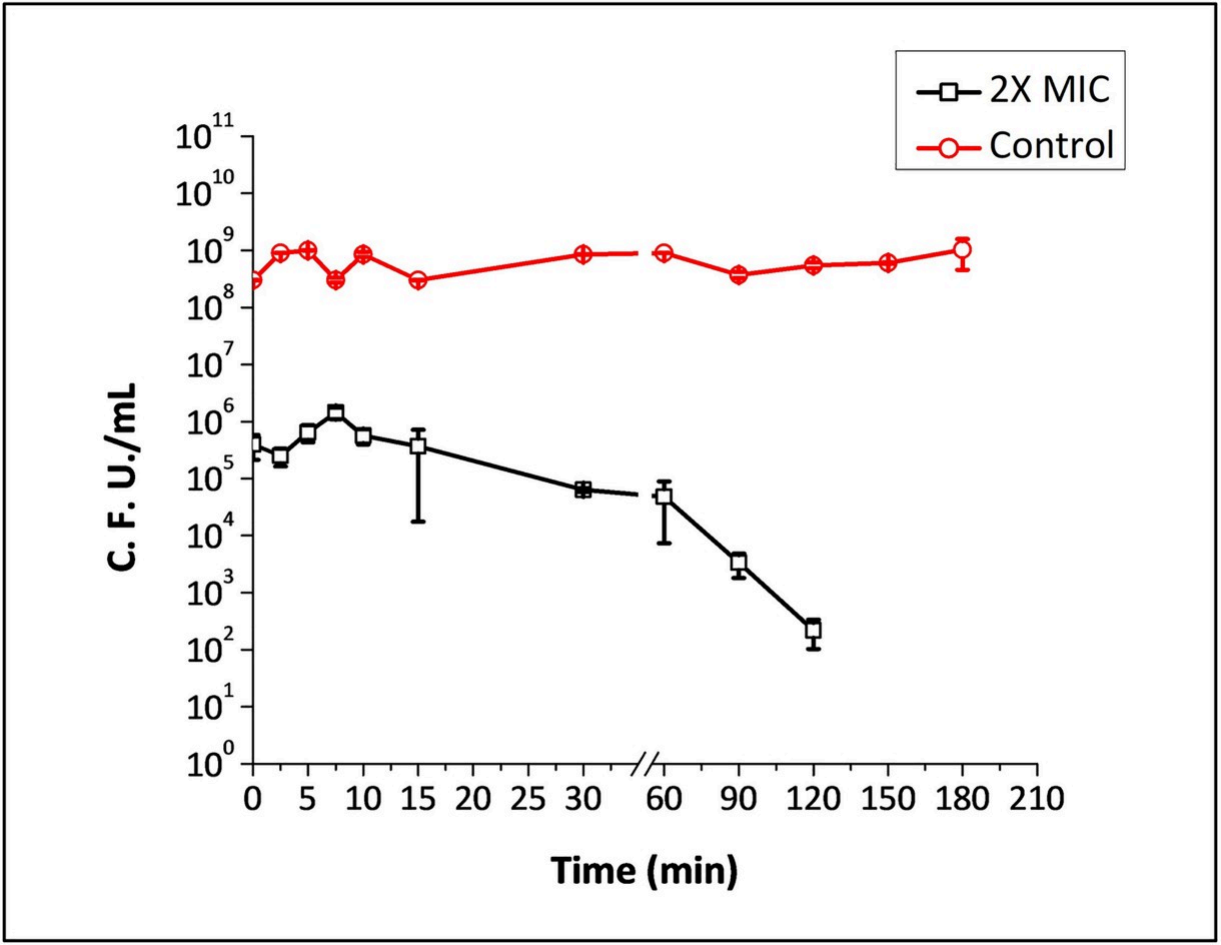
Time dependent killing of stationary phase planktonic cells of PAW1 against 2X MIC of acetic acid treated for 3 h.

Treatment of stationary phase planktonic cells of PAW1 with 2X and 4X MIC of acetic acid showed rapid killing over a period of time (Fig 3). 3 log_10_ cells of PAW1 were killed at 2X MIC of acetic acid as soon as the culture was exposed to it. The number of C.F.U. remained constant over 60 minutes of exposure to acetic acid following which there was a steady decline leaving no survivors after 3 h. 4X MIC was able to immediately eradicate the entire population leaving no survivors, demonstrating the antipersister activity of acetic acid.

**Fig 4:**
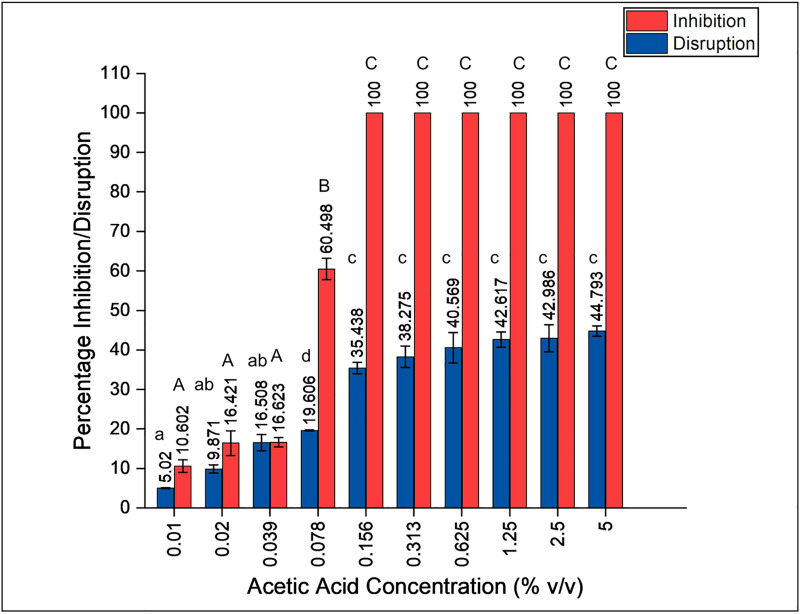
Effect of acetic acid on biofilm formation and disruption of PAW1. Percent inhibition of biofilm was determined at various concentration of acetic acid after 24 h. Percent disruption of 24 h old preformed biofilm was determined against various concentrations of acetic acid after 5 min of treatment. Different letters on the bars indicate that mean values of treatments are significantly different at p<0.01 according to Tukey’s post hoc test.

Biofilm inhibition assay demonstrated 100% inhibition of PAW1 at 0.156% of acetic acid (MIC value) after 24 h (Fig 4). Disruption of a 24 h old biofilm showed a dose dependent increase (p<0.01) in disruption ability of acetic acid (5 minutes treatment time) against PAW1 ranging between 5.02–44.79% (Fig 4).

**Fig 5:**
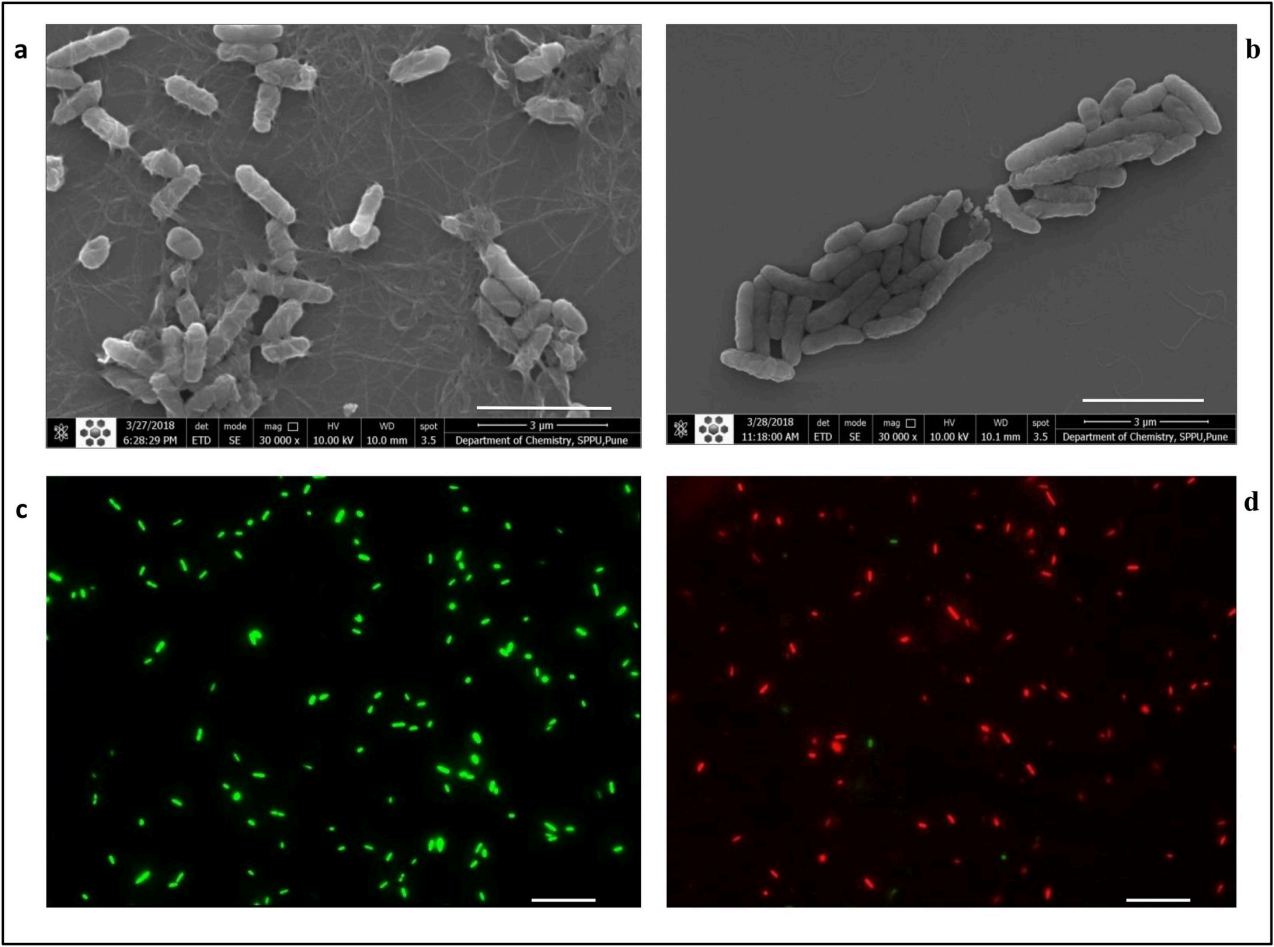
FESEM (at 30,000X magnification) and fluorescence microscopy (at 1000X magnification) of planktonic cells of PAW1. FESEM of untreated PAW1 cells with extracellular matrix (a) and reduced numbers of acetic acid treated PAW1 cells showing shrunken cells with disrupted extracellular matrix (b). Fluorescence images of the same samples at 528 nm (green) for SYTO9 signal, 645 nm (red) for PI signal were merged. Viable cells (green) in untreated sample (c) and acetic acid treated sample shows dead cells (red) with very few live cells (green) (d). Bar lines in (a) and (b) indicate 3 μm while in (c) and (d) indicate 5 μm.

**Fig 6:**
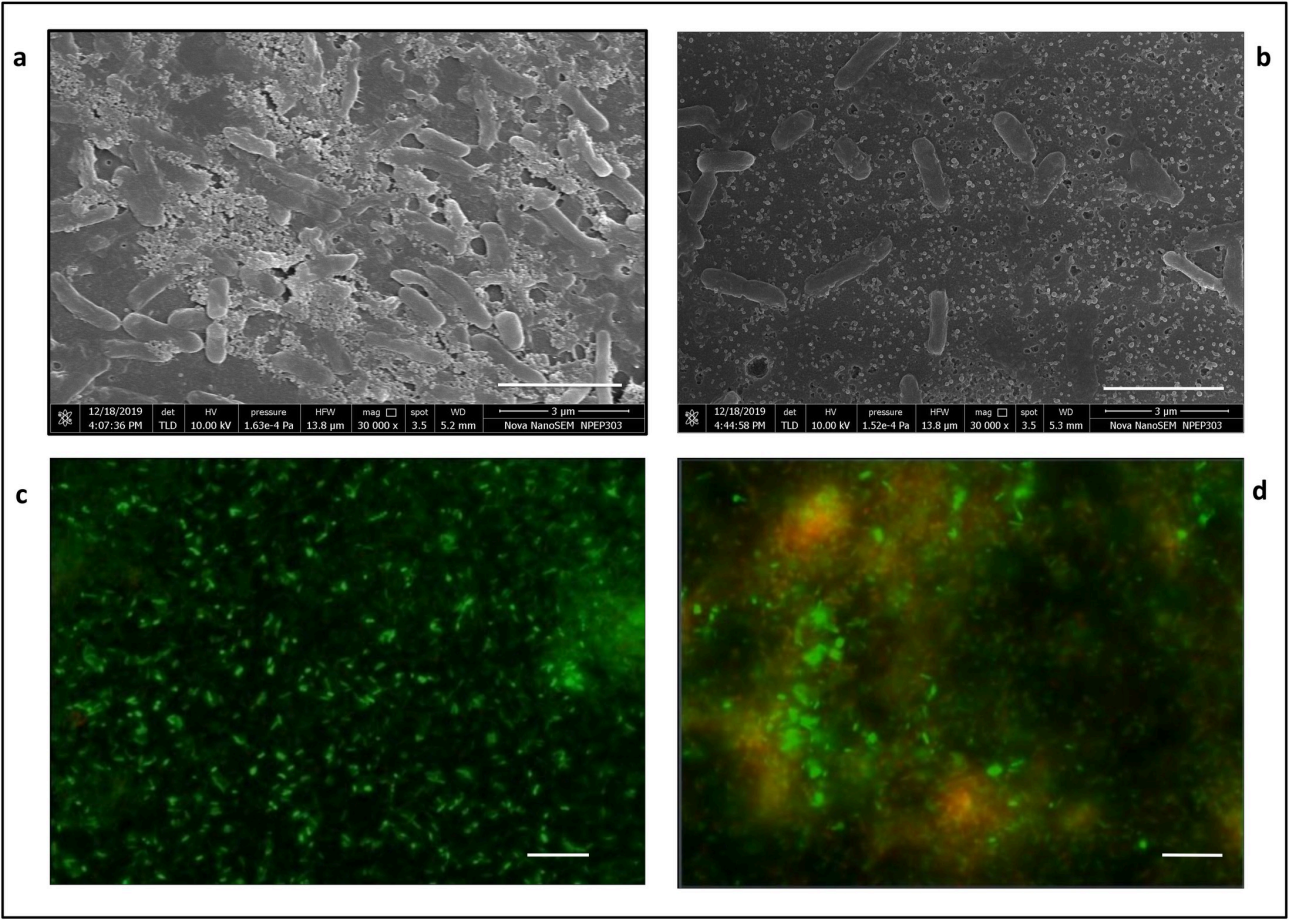
FESEM (at 30,000X magnification) and fluorescence microscopy (at 1000X magnification) of biofilm formed by PAW1. FESEM of untreated biofilm with extracellular matrix (a) and reduced numbers of acetic acid treated cells with disrupted extracellular matrix (b). Fluorescence images of the same samples at 528 nm (green) for SYTO9 signal and 645 nm (red) for PI signal were merged. Viable cells (green) are more in untreated sample (c) as compared to acetic acid treated sample (d) which also shows yellow red fluorescence of dead cells with a disrupted matrix. Bar lines in (a) and (b) indicate 3 μm while in (c) and (d) indicate 5 μm.

FESEM images of planktonic cells and biofilms of PAW1 showed a greater number of cells along with extracellular matrix in untreated control as compared to their reduced numbers and disrupted extracellular matrix in the sample treated with 0.625% acetic acid for 5 minutes (Figs 5 and 6). FESEM images of PAW1 after 5 min acetic acid treatment also showed shrinkage in cell size which was calculated by analyzing the area of all cells from each, control and treated sample images, using ImageJ software. Minimum/Maximum average area for control and treated cells was 6.69/15.85 and 5.13/15.50 respectively. Average absolute area for control and treated cells was 10.42±2.27 and 8.43±2.35 respectively. 19.04% (significant difference, p<0.01) shrinkage in cell size of acetic acid treated cells was observed along with disruption of extracellular matrix as compared to untreated control showing intact PAW1 cells with extracellular matrix (Fig 5A and 5B). BacLight™ live dead staining revealed dead cells in the acetic acid treated samples at the same dosages. Merged images of fluorescence microscopy for planktonic cells (Fig 5) and biofilms (Fig 6), obtained at 528 nm (green) for SYTO9 signal and 645 nm (red) for PI signal. The number of viable cells (green) was more in untreated sample as compared to acetic acid treated sample, showing dead cells (red) in planktonic forms; and yellow red fluorescence indicating presence of dead cells seen in acetic acid treated biofilm with a fuzzy disrupted matrix.

## Discussion

*P*. *aeruginosa* is reported as the most common pathogen (25% occurrence) associated with deep sternal wound infections [28]. Extensively drug resistant (XDR) and pan-drug resistant (PDR) strains of *P*. *aeruginosa* are a major therapeutic challenge in such chronic infections. It is imperative to understand how virulence factors play an important role in the infection dynamics of this pathogen based on which alternative treatment strategies can be proposed. There are very few reports on detailed characterization of XDR and PDR strains of *P*. *aeruginosa* from the Indian subcontinent. The present study characterizes an XDR *P*. *aeruginosa*, (designated as PAW1) isolated from the pus wound of a patient with recurrent sternal wound infection. Patient history suggested that this pathogen was resistant to most of the antibiotics used in empirical therapy and therefore was selected as an attention-grabbing candidate for study. The study further explored the possible alternative treatment strategies which can be proposed to treat such XDR pathogens.

Any organism is characterized as XDR owing to its non-susceptibility against at least one agent from all but two or fewer antimicrobial categories. In order to be characterized as PDR, an isolate must be resistant to all approved antimicrobial agents [27]. Antibiotic resistance studies displayed that PAW1 was an XDR isolate showing resistance to all classes of antibiotics including carbepenems, cephems, β-lactam inhibitor combinations and fluroquinolones with MICs ≥1024 μg/mL, the only exception being gentamicin and netilmicin from aminoglycosides group and polymyxin B from lipopeptides group with MIC of ≤2 μg/mL.

PAW1 was found to express virulence factors such as haemolysins, caseinase, gelatinase and lipase suggesting its ability to induce tissue damage. Furthermore, this isolate also harboured the ability to produce siderophores, which may render it able to survive and evade the host immune system. Biofilm formation is another major factor contributing towards the pathogenicity of *Pseudomonas* spp. Biofilms serve as a physical barrier against antibiotics and the host immune system [29]. Moreover, dormant persister cells that are tolerant to antibiotics are protected by the biofilm matrix and are a cause for recalcitrant infections which are difficult to treat [23, 30]. PAW1 was found to be a strong biofilm producer and also showed the ability to form dormant persister cells in presence of gentamicin and polymyxin B.

Antibiotic tolerant persister cells have been blamed to cause recalcitrant infections that are difficult to treat [30]. Since PAW1 was isolated from a chronic sternal wound infection that was untreatable using any antibiotic regime, the possibility of presence of persister cells was explored. 100X MIC of gentamicin and polymyxin B revealed a population of persister cells, whose metabolic dormancy probably did not allow for complete eradication of infection. To the best of our knowledge there are no previous reports demonstrating *P*. *aeruginosa* persisters in presence of polymyxin B. Gentamicin has not been studied for persister formation in *P*. *aeruginosa*. However, Mlynarcik and Kolar (2017) have studied the formation of persister cells in *P*. *aeruginosa* PAO1 against various antibiotics including the aminoglycoside, tobramycin and polymyxin B [31]. They demonstrate that treatment of tobramycin (at 104 μg/mL, 80X MIC) resulted in complete killing of PAO1 at 24 h; while our study revealed that treatment of gentamicin (at 100 μg/mL, 100X MIC) resulted in relatively large population of PAW1 persisters at 24 h. Similarly, treatment of PAO1 with polymyxin B (at 64 μg/mL, 80X MIC) showed regrowth of the cells after 24 h, possibly due to mutation [31]; whereas our study showed a typical biphasic killing curve indicating the presence of persisters in presence of polymyxin B (at 2.5 μg/mL, 100X MIC). These comparisons however cannot be held conclusive due to the difference in concentration of antibiotics applied. The ability to form biofilms and to form tolerant persister cells in presence of gentamicin and polymyxin B could possibly be responsible for the recurrent infection history seen in the patient.

Based on the virulence and antibiotic resistance assays, as well as noting the patient history of not responding to any antibiotic treatment, it was concluded that, alternative treatment options to inhibit or kill PAW1 need to be explored. Use of forgotten antimicrobials and antiseptics are gaining interest in infected wound management and for treatment of infections caused by multidrug resistant pathogens [32–34]. The use of acetic acid as a topical agent has been reported for the treatment of superficial wounds infected by *P*. *aeruginosa* [13, 15, 16, 35]. The present study demonstrates antimicrobial, antipersister and antibiofilm potential of acetic acid against this XDR isolate for topical application.

MIC of acetic acid against PAW1 was found to be 0.156% which is in accordance with previous reports [17]. Microscopy of PAW1 and cell viability assays confirmed that treatment of cells with acetic acid (0.625%, 5 min) caused cell shrinkage (19.04%) and cell death. Bjarnsholt et al., have demonstrated that pH of acetic acid below 4.76 is required to be effective against *P*. *aeruginosa* biofilms [18]. It has also been reported that toxicity of weak acids such as acetic acid towards bacterial cells is not only pH dependent but the protonated form of acetic acid diffuses through the cell wall, causing disruption of the proton gradient, thus resulting in bacterial killing [17, 36]. FESEM images in the present study revealed shrunken PAW1 cells on treatment with acetic acid, possibly indicating membrane damage due to the disruption of proton gradient.

Treatment with 2X and 4X MIC of acetic acid caused rapid killing of PAW1 cells over a period of time causing instantaneous killing at 4X MIC. However, in the MTT assay, we observed 2.11% survivors at 0.625% (4X MIC) of acetic acid treated for 5 min and tested. It should be noted that, the MTT assay, used for cytotoxicity study, is an indirect method of measuring viability based on the reduction of tetrazolium dye by the cellular metabolic activity due to the enzymes and cofactors. The percent viability seen at higher concentrations of acetic acid using MTT assay may be due to the enzymes that would still be functional at lower rates, thus being able to reduce the MTT dye.

However, as we wanted to compare the cytotoxic effect of acetic acid on PAW1 as well as on L929 cell, it was thought necessary to use the same method of analysis to compare the percent viabilities. MTT assay was therefore performed using various concentrations and time points to determine the most appropriate concentration of acetic acid to be selected for confirmatory studies.

On the other hand, the time kill assay (at 2X and 4X MIC of acetic acid) was estimated by determining the colony forming unit of the surviving PAW1 population. The C.F.U. counts clearly reveal that all PAW1 cells showed a ~3 log_10_ reduction within 2.5 min at 2X MIC (0.33%) and were killed instantaneously at 4X MIC (0.625%) of acetic acid. The ability of acetic acid in clearing off the bacterial load within a short period not only proves to be an effective treatment strategy but also greatly decreases the possibility of antibiotic resistance evolving during otherwise extended treatments associated with chronic infections [37]. Acetic acid has been reported to be an effective treatment strategy when used alone or in combination with other antibacterial agents, such as tobramycin, ciprofloxacin, and colistin to treat *P*. *aeruginosa* infections [18]; further recommending the need to explore if persisters cells were formed using such combinations. Our study is probably the first report showing the efficacy of acetic acid at low concentrations (0.625%, 4X MIC) to cause complete killing of cells leaving no persisters.

There are reports on biofilm inhibition and disruption ability of acetic acid. Halstead et al., reported that ≤0.31% acetic acid is required for complete inhibition of biofilm formation by all isolates of *P*. *aeruginosa* tested [17]. Our study supports this finding with complete inhibition observed at 0.156% acetic acid against newly isolated XDR strain of *P*. *aeruginosa* PAW1. Complete eradication of preformed *P*. *aeruginosa* PAO1 biofilm has been reported at low dosages of 0.3–0.5% acetic acid treated for long duration up to 24 h [17, 18]. Biofilm disruption in case of PAW1 was studied at various concentrations of acetic acid treated for 5 min duration. PAW1 biofilm could not be completely eradicated even at a concentration of 5%. Preformed biofilm of PAW1 treated at 0.625% acetic acid for 5 min resulted in 40.57% disruption. Microscopy of biofilms treated at this dosage showed reduction in biofilm mass and cell death.

*In vitro* toxicity tests are usually carried out using host cells that are homologous with the human tissue concerned. Both fibroblasts and keratinocytes play a major role in maintaining the skin barrier and also play an important role in immune response, inflammatory process and wound healing [38]. Therefore, L929 mouse fibroblast cell line was selected for the cytotoxicity assays. Earlier studies have reported that acetic acid is toxic to fibroblast cells when studied *in vitro* [39]. *In vivo* studies have also shown promising results of acetic acid in treatment of bacterial infections [40, 41]. A case study and a randomized clinical trial has proved the efficacy of 1% acetic acid to treat chronic wounds infected with *Pseudomonas* spp. [35, 42]. Other studies also support the efficacy of 1–5% of acetic acid against *P*. *aeruginosa* infection, which did not respond to conventional therapies, without any adverse effect on host tissue cells [15, 16, 41]. Our study reveals that five minutes treatment of acetic acid had an IC_50_ of 0.625% against L929 host cells at which more than 90% of the PAW1 cell population was killed. It can thus be suggested that, repeated treatment of infected wounds using this dosage may be effective in completely killing all PAW1 cells and may still allow surviving fibroblast cells to regenerate. It should be noted that this dosage was also effective in killing stationary phase planktonic PAW1 cells leaving no persisters and at the same time was less toxic to L929 mouse fibroblast cells. However, a greater number of isolates need to be tested to be confirm these findings. It can also be suggested that, repeated treatment of infected wounds using this dosage may be effective wound management strategy which however needs further evaluation using *in vivo* models. Our findings also provide useful information for cleaning of hospital equipment and contaminated surfaces.

## Conclusion and future prospects

This study highlights a newly isolated extensively drug resistant *P*. *aeruginosa* PAW1, which showed the ability to form persisters against 100X MICs of gentamicin and polymyxin B that is not previously reported. The study uses this newly isolated PAW1, to demonstrate the antibacterial, antipersister and antibiofilm effects of acetic acid and compare the same with previous studies carried out using standard isolates such as *P*. *aeruginosa* PAO1. It would also be interesting to further study PAW1 using whole genome sequencing to understand the diversity of resistance genes it harbours, and also to understand if biofilms of PAW1 harbour increased number of persister population.

## Supporting information

S1 Table Effect of acetic acid on percent survival of L929 cell line. (DOCX)

S2 Table Effect of acetic acid on percent survival of PAW1 cells. (DOCX)
