# Supplementary material for: Antibiofilm and antipersister activity of acetic acid against extensively drug resistant Pseudomonas aeruginosa PAW1
Source: PLoS One. 2021 Feb 2;16(2):e0246020. doi: 10.1371/journal.pone.0246020 (PMC7853517; doi:10.1371/journal.pone.0246020)
Supplement: S1 Table — (DOCX) [file pone.0246020.s001.docx]

**S1 Table: Effect of acetic acid on percent survival of L929 cell line**

| **Acetic Acid %**  **(v/v)** | **Percent survival of L929 cell line (Mean ±SD)** | | | |
| --- | --- | --- | --- | --- |
|  | **2.5 min** | **5 min** | **7.5 min** | **10 min** |
| **5** | 2.65±0.12 | 1.52±0.11 | 0.88±0.84 | 0.96±0.82 |
| **2.5** | 2.32±0.21 | 1.84±0.02 | 1.54±0.80 | 1.12±0.75 |
| **1.25** | 4.01±0.80 | 2.18±0.01 | 2.07±1.41 | 1.42±0.08 |
| **0.625** | 59.47±2.25 | **49.76±0.89** | 2.20±0.10 | 1.84±0.24 |
| **0.313** | 81.55±0.79 | 68.97±0.17 | 35.45±0.06 | 20.02±0.95 |
| **0.156** | 90.90±0.61 | 80.47±1.91 | 60.21±4.58 | 58.09±1.54 |
| **0.078** | 97.76±4.01 | 95.10±0.29 | 90.91±3.91 | 87.55±0.38 |
| **0.039** | 99.14±0.82 | 98.32±1.96 | 98.43±0.40 | 94.81±1.27 |
| **Control** | 100 | 100 | 100 | 100 |

Results of all experiments were determined as means± SD. Statistical analysis was done using one-way ANOVA followed by Tukey’s HSD post hoc test. Differences between all time points for each concentration were considered statistically significant at p<0.01. Percent survival of L929 at acetic acid concentrations 0.625% and 0.156% was statistically significant (p<0.01) between the various treatment times. Percent survival of L929 at acetic acid concentrations ≥1.25% (ranged between 1.42±0.08 and 4.01±0.80) and ≤0.078% (ranged between 87.55±0.38 and 99.14±0.82) was statistically insignificant.
