# Supplementary material for: Antibiofilm and antipersister activity of acetic acid against extensively drug resistant Pseudomonas aeruginosa PAW1
Source: PLoS One. 2021 Feb 2;16(2):e0246020. doi: 10.1371/journal.pone.0246020 (PMC7853517; doi:10.1371/journal.pone.0246020)
Supplement: S2 Table — (DOCX) [file pone.0246020.s002.docx]

**S2 Table: Effect of acetic acid on percent survival of PAW1 cells**

| **Acetic Acid % (v/v)** | **Percent survival of PAW1 cells (Mean ±SD)** | | | |
| --- | --- | --- | --- | --- |
|  | **2.5 min** | **5 min** | **7.5 min** | **10 min** |
| **5** | 2.78±0.41 | 1.09±0.01 | 0.68±0.22 | 0.24±0.15 |
| **2.5** | 4.20±0.79 | 1.15±0.13 | 0.90±0.32 | 0.78±0.32 |
| **1.25** | 4.04±0.92 | 1.39±0.07 | 1.36±0.46 | 0.95±0.79 |
| **0.625** | 62.78±3.80 | **2.11±0.20** | 1.68±0.07 | 1.60±0.33 |
| **0.313** | 91.33±0.90 | 78.82±1.06 | 55.36±2.72 | 3.45±0.38 |
| **0.156** | 97.95±1.71 | 95.21±0.33 | 80.73±0.96 | 73.38±2.08 |
| **0.078** | 99.28±1.27 | 98.12±2.63 | 96.78±0.04 | 94.98±0.68 |
| **0.039** | 100.91±2.76 | 99.49±1.30 | 97.44±0.02 | 98.88±0.55 |
| **Control** | 100 | 100 | 100 | 100 |

Results of all experiments were determined as means± SD. Statistical analysis was done using one-way ANOVA followed by Tukey’s HSD post hoc test. Differences between all time points for each concentration were considered statistically significant at p< 0.01. Treatment with acetic acid concentrations between 0.03 and 0.625% caused statistically significant difference (p<0.01) in percent survival of PAW1 at various treatment times. Percent survival of PAW1 at acetic acid concentrations ≥1.25% (ranged between 0.24±0.15 to 4.20±0.79) and ≤0.078 % (ranged between 94.98±0.68and 100.91±2.76) was statistically insignificant.

5 min treatment at 0.625% acetic acid showed maximal killing (98%, p<0.01) of PAW1 cells with moderate toxicity against L929 cells (~50 %, p<0.01; Refer Table S1) and was therefore chosen for further studies.
